# Guiding gaze via gaze: Effect anticipation during intentional saccadic gaze leading

**DOI:** 10.1007/s00426-025-02231-z

**Published:** 2026-02-14

**Authors:** Eva Landmann, Lisa Weller, Eva Riechelmann, Wilfried Kunde, Lynn Huestegge

**Affiliations:** Department of Psychology III, University of Würzburg, Würzburg, Germany

## Abstract

Our actions often evoke foreseeable behavioral responses in others. For instance, fixating an object can trigger another person to look toward the same location. Ideomotor frameworks assume that one's own actions are initiated by anticipating this evoked behavior of another person. However, in gaze leading situations, a partner’s gaze following is initially discernible only peripherally, raising the question of whether corresponding anticipations still affect the gaze leader’s eye movements. This potentially important mechanism underlying gaze interaction has not yet been explicitly examined. We conducted two experiments using a novel adaptation of the response-effect compatibility (REC) paradigm. Within a card-game cheating scenario, participants performed a saccade to one of two target objects (card decks). Contingent upon this saccade, an on-screen face foreseeably looked to the same object (compatible) or to the opposite side (incompatible), a perceptual consequence of the participant’s saccade that was initially only detectable in peripheral vision. Crucially, participants initiated saccades significantly faster in the compatible compared to the incompatible condition, but only in an experimental setting without additional constraints preventing direct fixation of the action effect. This suggests that participants anticipated their (virtual) partner’s gaze responses, which in turn either facilitated or hampered the production of their own eye movements. This finding aligns with the idea that basic ideomotor processes also underlie social gaze leading/following scenarios. Consistent with previous REC studies in the non-social domain, the results also indicate that the effect is sensitive to specific experimental settings and may disappear when cognitive demands are too high or when the interaction situation is less realistic.

## Public significance statement

In social settings, actions often elicit predictable responses from others, and research shows that anticipating these effects is relevant even for basic action control. Our study demonstrates that this basic cognitive principle can extend to the control of eye movements during gaze interactions: Under certain conditions, participants initiated gaze shifts towards objects faster when another person foreseeably followed their gaze. These findings broaden our understanding of action control with respect to another person’s (gaze) behavior.

## Guiding gaze via gaze: effect anticipation during intentional saccadic gaze leading

Humans move their eyes almost constantly to perceive new information. In this process, each saccade is associated with an immediate effect, namely the change of the retinal image. For instance, when moving the eyes to look at a specific object (e.g., a face), the immediate effect of the saccade is the representation of this object in the fovea. Recent studies have suggested that this anticipated effect is crucially involved in the generation of saccades (Herwig & Horstmann, 2011; Huestegge & Kreutzfeldt, 2012; Riechelmann et al., 2021), well in line with an ideomotor (effect-based) view of action control.

### Ideomotor framework and saccade control

The ideomotor framework aims at explaining how perceptual goals are translated into specific body movements (for reviews see e.g., Hommel, 2013; Pfister, 2019; Shin et al., 2010). The approach rests on the fact that almost all our body movements inevitably produce some kind of sensory effects. A knock on the table, for instance, results in a knocking sound, a certain tactile feeling, and a visual impression of the knocking motion. The basic idea of the ideomotor framework is that people acquire bidirectional associations between specific motor activity and the resulting sensory effects. Since it is assumed that the associations are bidirectional, they can be used for action control. That is, an anticipation of a certain sensory effect (e.g., a knocking sound) should automatically activate (to some extent) the associated motor pattern, thereby enabling its efficient execution. This idea is supported especially for manual actions (e.g., Ansorge, 2002; Camus et al., 2018; Elsner & Hommel, 2001, 2004; Janczyk & Lerche, 2019; Kunde, 2001; Kunde et al., 2004; Riechelmann et al., 2019; Wolfensteller & Ruge, 2011), while evidence for some other motor domains and effect features, for example, vocal actions and semantic features, is rather mixed (Koch et al., 2021).

Studies on saccade control have investigated whether this ideomotor approach also applies to eye movements. For example, it has been shown that anticipatory saccades towards future effect locations can serve to monitor action effects induced by manual actions (Pfeuffer et al., 2016, 2023). However, there is also research addressing the issue of whether eye movements themselves may be driven by anticipated effects. To this end, Huestegge and Kreutzfeldt (2012) asked participants to react to lateralized auditory stimuli with a corresponding saccade to a left or right object (e.g., a square on the left side and a rhombus on the right side). Importantly, prior to the auditory stimulus, one of the target objects (i.e., a square or a rhombus) was presented at central fixation. This object could be congruent with the peripheral saccade target (e.g., a square when the lateralized tone required the execution of a saccade to the left, where a square was shown) or incongruent (e.g., a rhombus when the lateralized tone required a saccade to the left, where a square was shown). According to the ideomotor framework, the congruency manipulation should influence participants’ saccade latencies (i.e., the time between the auditory stimulus and saccade initiation). That is, participants repeatedly experienced that a saccade to one side (e.g., left) triggers a certain effect (e.g., foveation of a square) and should thus acquire bidirectional associations between the saccade and the corresponding effect. The presentation of the effect prior to the auditory stimulus should then automatically activate the corresponding saccade. This is beneficial if the auditory stimulus requires the same action (congruent condition), but not if it requires the opposite action (incongruent condition). In line with these assumptions, participants initiated saccades faster in the congruent compared to the incongruent condition, suggesting that anticipated action effects indeed play a crucial role in basic saccade control, similar to the manual control domain (see also Herwig & Horstmann, 2011; Riechelmann et al., 2017; Verschoor et al., 2013).

In the context of effect-based saccade control, it is also important to highlight the crucial role of trans-saccadic integration. When integrating the information of the same object that is first encountered parafoveally before being eventually fixated, it has been shown that learning mechanisms are at play that assist the control of saccadic programming (e.g., in the case of saccadic adaptation, see Valsecchi et al., 2020) and the prediction of how peripheral objects will appear once they are foveated. Such predictions even affect the way we perceive the visual periphery in general, thereby contributing to the perception of a stable, well-resolved visual world (Herwig & Schneider, 2014; Valsecchi et al., 2020). Together, these phenomena emphasize how ideomotor theory also underlies basic trans-saccadic integration phenomena. 

A crucial difference between manual actions (the focus of most ideomotor control studies) and oculomotor actions is the fact that manual actions are typically used to interact with and manipulate objects in the environment (e.g., knock on a door, type on a computer, open a window), whereas oculomotor actions are mainly executed to move specific information into the visual field, but not to manipulate objects external to the observer. Only in the last few decades, gaze-controlled technology has put eye movements as means to control the environment more into focus (e.g., Esteves et al., 2015; Ramesh & Rishikesh, 2015; Zhang et al., 2013). In social situations, however, eye movements are rather common means to influence other people’s behavior, thus suggesting that social situations may be particularly interesting for studying effect-based saccade control processes.

### Eye movements in social interactions

Eye movements seem pivotal for human communication, and humans are highly sensitive to the gaze of others (e.g., Emery, 2000; Kano & Call, 2014). That is, if another person is looking at a certain point in space, people tend to instantaneously orient their attention toward the direction of the other person’s gaze. This is known as *gaze cueing/following* (for a review see Frischen et al., 2007; McKay et al., 2021), and it allows the gaze leader (i.e., the person initially looking at a certain point in space) and the gaze follower to orient their attention to the same object without any additional speaking or gesturing. The ability (and tendency) to follow another person’s gaze seems to develop early in life and enables the gaze follower to draw conclusions about the goal of the gaze leader or even to predict future actions (Farroni et al., 2004; Hood et al., 1998; Scaife & Bruner, 1975). Gaze cueing studies have suggested that following another person’s gaze (by overtly looking at the same location or by covertly shifting attention to that location) happens rapidly and reflexively (e.g., Driver et al., 1999; Friesen & Kingstone, 1998, 2003).

Importantly, however, the fact that humans follow gaze so consistently also has benefits for the gaze leader, who can use gaze to communicate and direct the follower’s attention, that is, to actively control the behavior of others. From the gaze leader’s point of view, gaze following is a rather consistent and foreseeable effect of their own action, for which they tend to experience a sense of agency (i.e., an impression of being the cause of the other’s gaze shift; Brandi et al., 2019; Stephenson et al., 2018). Given this consistency, ideomotor reasoning would suggest that these and similar social consequences should be able to shape action control (in this case saccade control) via the same basic mechanisms of ideomotor control that operate in non-social contexts: When an agent’s action reliably elicits behavioral responses in other humans, associations form between the motor patterns and the corresponding social effects. Anticipating these effects can then activate the associated motor pattern. This extension of ideomotor theory to social outcomes has been termed *sociomotor action control*, emphasizing that anticipated action effects can include another person’s behavior rather than only inanimate changes (Kunde et al., 2018).

Supporting the sociomotor framework, several studies have shown that social action effects (i.e., any behavior of another person that is consistently triggered by our own motor activities), can be used for action control similar to inanimate action effects (e.g., Flach et al., 2010; Kunde et al., 2011; Lelonkiewicz et al., 2020; Müller, 2016; Müller & Jung, 2018; Pfister et al., 2013; Weller et al., 2019, 2020), both for manual actions but also for saccade control (Herwig & Horstmann, 2011; Riechelmann et al., 2021). For instance, in the study by Herwig and Horstmann (2011) participants sat in front of a computer screen and moved their eyes from a central fixation point to the left or right, where two faces with a neutral emotional expression were displayed. Upon fixation of the face, it changed facial expression (from neutral to happy or angry, depending on saccade direction). Results showed that the vertical landing position of the saccade was influenced by the face’s subsequent expression: Saccades would hit the neutral face more towards the region where most of the physical change would occur, that is, at a lower vertical position (more towards the mouth region) when moving to a face that would change to a happy (vs. angry) expression. This result suggests that participants anticipated the subsequent reaction of the face stimulus and that this anticipation influenced the parametrization of the saccade (Herwig & Horstmann, 2011). In another study, participants were asked to direct their gaze to one of two on-screen faces (see also Grossheinrich et al., 2018, 2022) which initially appeared in the periphery with closed eyes and, once looked at, responded with either direct or averted gaze (Riechelmann et al., 2021). Participants executed saccades more quickly when primed by a centrally presented face whose gaze behavior matched that of the subsequent target face, compared to when the prime was incongruent. This suggested that participants anticipated whether the gaze of the on-screen faces would respond to their gaze with averted or direct gaze, indicating that effect anticipation also plays a role in gaze interaction.

All of these previous studies have focused on situations in which the gaze-initiated social action effect (i.e., the other person’s behavioral response) was immediately visible in *foveal* vision. This emphasis is understandable, given that socially relevant signals are often processed foveally and that foveal input plays a key role in tasks such as object recognition (Stewart et al., 2020). However, this setup fundamentally differs from typical gaze leading situations, in which the partner’s gaze following is initially perceived in peripheral or parafoveal vision: Although gaze leading may begin with eye contact, the leader’s saccade toward a new location means that the follower’s subsequent gaze shift necessarily occurs outside the foveal area. This distinction is critical when considering whether and how anticipating another person’s gaze following might activate corresponding eye movements toward the shifted-to location, because the desired effect is not the foveation of a particular object but rather the perception of the partner’s gaze shift in the visual periphery. Consequently, the question of whether basic ideomotor mechanisms operate in gaze leading remains unresolved.

### The present study

In the present study, we wanted to investigate whether the expected gaze behavior of another person would influence saccade control in a gaze leading scenario, in which participants were able to guide a (virtual) interaction partner’s gaze in different directions through their own eye movements. This is particularly interesting as, in this setting, the follower’s gaze shift (i.e., the onset of the action effect) occurred peripheral to the participant’s saccade target position (i.e., the effect did not appear at the saccade landing position). Thus, for the first time we here address the situation of how we direct another person’s gaze with our own gaze towards specific spatial locations from an ideomotor perspective.

Ideomotor theory in a social context (Kunde et al., 2018; see also French & Raven, 1959; Anderson et al., 2012; Paulus, 2025) proposes that action control involves bidirectional associations between an action and its social effect, allowing corresponding actions to be triggered by anticipating these effects. This mechanism has been demonstrated in a growing number of studies for saccades, but with a significant limitation: Action effects were presented in central vision. This could potentially facilitate ideomotor mechanisms but does not reflect important real-world scenarios of social action control such as gaze leading/following. To address this limitation and to test sociomotor theory more rigorously, we developed a novel, rather complex gaze interaction paradigm in which the action effect (the gaze follower’s response to the participant’s own gaze) initially appeared in peripheral vision, as is typically the case in real life. If the gaze follower’s reactions influence the gaze leader’s saccade control, this would provide more compelling support for the role of effect anticipation in these important instances of social action control. Conversely, absence of such effects (due to, for example, perceptual or attentional limitations in parafoveal or peripheral vision) might indicate constraints on ideomotor mechanisms outside experimental settings optimized for saliency and other facilitating factors (such as highly resolved foveal processing).

To achieve some extent of ecological validity, we aimed to construct a task that represented a (somewhat) realistic gaze interaction by transferring a response-effect compatibility (REC) setup (Ansorge, 2002; Janczyk et al., 2023; Kunde, 2001; Rieger, 2007; Yamaguchi & Proctor, 2011) to the social domain. In particular, participants sat in front of a computer screen on which the face of another person was displayed. Participants were instructed to *guide* the gaze of the other person to a certain object (card decks displayed in the left and right half of the screen) by moving their eyes to one of the decks. Each participant’s saccade triggered a gaze shift of the on-screen face. In different blocks, the gaze shift of the central on-screen face was either spatially compatible with the participant’s saccade (i.e., gaze following behavior, compatible action-effect mapping) or incompatible (i.e., gaze to the opposite side, incompatible action-effect mapping). Thus, the subsequent on-screen gaze shift was objectively anticipable for participants.

We embedded this task in a cover story of a casino setting. In brief, participants played the role of an expert cheater who would teach a student (i.e., the on-screen face) how to cheat at a card game. To that end, participants should signal the student which card would be relevant next by moving their eyes to a specific card deck following pre-agreed rules. The casino scenario was chosen to make gaze-based communication feel purposeful and compatible and incompatible gaze responses equally plausible (i.e., changing signaling strategies to avoid detection). Although the cover story involved a minor ethical transgression, we framed it as a teacher-student interaction and de-emphasized illegal aspects to maintain participant engagement while minimizing potential negative influences. To ensure that participants attended to the student’s gaze responses we implemented catch trials (see Pfister et al., 2017, for a similar approach), in which the student (the on-screen face) would violate the current mapping rule. Furthermore, we implemented no-go trials (in which participants were asked to withhold any action) to prevent pre-selected or stereotypic response decisions. In contrast to other paradigms (see above), our study introduced the onset of the action effect (i.e., the follower’s gaze shift) *in the parafovea/periphery* of the participant’s fixation position while ensuring that relevant information was still perceived.

We expected compatibility-related effects in saccadic reaction times (RT), that is, increased saccade latencies for an incompatible (vs. compatible) action-effect mapping similar to previous REC effects reported throughout the literature for social environments (e.g., the production of facial expressions, Kunde et al., 2011) as well as non-social environments (e.g., keypresses generating differently colored squares, Pfister et al., 2010). Such an increase in the incompatible condition would be in line with the idea that the anticipated effect of a required oculumotor response additionally activates the motor pattern associated with a spatially corresponding (but currently not required) response. That is, anticipating a gaze to the left (displayed by the other person) should activate a saccade to that same location, whereas in the incompatible condition the opposite saccade would be required. A compatibility-related RT effect thus suggests that anticipated action effects (here: in terms of the gaze direction of the gaze follower) are involved in action initiation within the oculomotor domain. Additionally, we expected that compatibility effects would grow in size with increasing RT, similar to REC effects in the manual domain. That is, we anticipated a larger difference between RTs for compatible and incompatible action-effect mappings in trials with slower responses, with the reasoning that slow-response trials provide more time for the endogenously activated effect codes to interfere with the codes of the action that is actually required (Pfister et al., 2014; Wirth et al., 2016).

We conducted two versions of the experiment: In one of them, Experiment B (“unrestricted gaze”), we did not implement a restriction on fixating on the action effect after its initiation. In Experiment A (“restricted gaze”), by contrast, we excluded any possibility for participants to return their gaze to the target face by masking the face whenever they tried to look at it. Note that in both experiments, the actual effect occurrence (gaze shift of the on-screen face) was initially experienced in the parafovea/periphery. Observing compatibility-related RT effects in either version of our experiment (differing in the degree of experimental control over fixations of the action effect) would support the hypothesized role of action-effect associations in saccade production in line with the sociomotor framework (Kunde et al., 2018), with slightly different implications regarding the conditions required. Experiment A was designed in a way to ensure that the crucial visual information is only available in the periphery to prevent any potential re-engagement with the target. In contrast, Experiment B established a more open and arguably more naturalistic environment in which participants were not completely restricted from glancing back at, that is, foveating the target face to monitor the action effect after its onset (even though they were still instructed not to). Comparing these two experiments allows us to explore the development and use of action-effect associations in gaze-leading scenarios with varying levels of experimental restriction.^1^

## Experiment A

In Experiment A (“restricted gaze”), we confined participants to perceiving the action effect solely via parafoveal/peripheral vision. If they attempted to re-fixate the target face after its gaze shift, the face vanished and was replaced by a warning message, limiting participants to the visual cues typically available immediately after leading an interaction partner’s gaze.

### Method

#### Transparency and openness

We report how we determined our sample size, all data exclusions (if any), all manipulations, and all measures in the study. The design and analysis of both experiments were preregistered. The preregistrations, along with all data and analysis scripts are openly available on the Open Science Framework (Experiment A: https://osf.io/vw3na; Experiment B: https://osf.io/4cebg). Data were analyzed using R, version 4.4.1 (R Core Team, 2024), and the package ‘afex’, version 1.4.1 (Singmann et al., 2024).

At the time the study was conceived and conducted, no ethics approval was required according to the ethical guidelines of the German Society for Psychology (Deutsche Gesellschaft für Psychologie) and regulations of the local ethics committee, as our study provided signed informed consent from study participants, collected data anonymously, did not involve any form of deception, and had no foreseeable negative impact on participants.

During the preparation of this work the authors used ChatGPT-4o (OpenAI, 2024) in order to improve the readability and language of the manuscript. The authors reviewed and edited the content as needed and take full responsibility for the content of the published article.

#### Participants

In total, 108 participants were recruited (data collection in 2021–2022). Four participants were excluded from the analysis because data collection had to be aborted due to technical issues. Data of the remaining 104 participants were analyzed (mean age = 24.0 years, *SD* = 3.9 years, age range: 18–37 years; 24 male). This sample size was determined a priori (power = 0.80, G*Power, Faul et al., 2007) based on the effect size observed in Experiment B (*d*_*z*_ = 0.28, note that data for Experiment A was collected after Experiment B). All participants gave informed consent before the experiment started and received either course credits or payment for compensation. Participants reported normal or corrected-to-normal vision and were naïve with respect to the purpose of the study. The study protocol and sample size were preregistered prior to data collection on the Open Science Framework (https://osf.io/a26fn).

#### Apparatus and stimuli

Participants performed the experiment in a dimly lit room where they were seated in front of a 20-in. cathode ray monitor with a spatial resolution of 1,024 × 768 pixels (refresh rate: 100 Hz). They viewed the screen from a distance of approximately 65 cm with a forehead and chin rest stabilizing the head. A video-based eye tracker (EyeLink1000, SR Research, Ontario, Canada) was used to track eye movements of the right eye at a sampling rate of 1,000 Hz. Manual responses were recorded on a standard computer keyboard. The experiment was programmed using Experiment Builder (SR Research, Ontario, Canada).

**Fig. 1:**
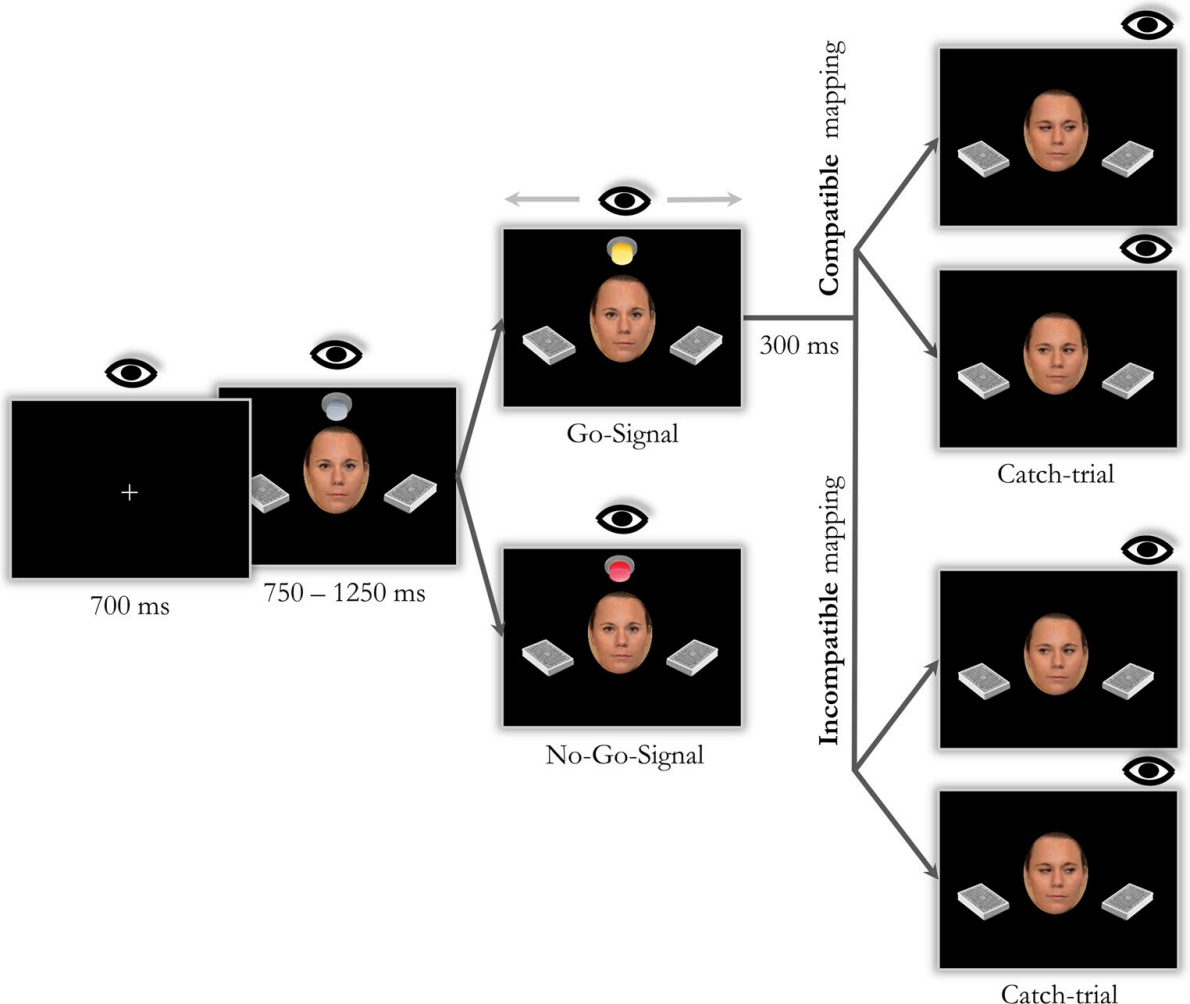
*Illustration of the Trial Structure in Experiments A and B. * After the presentation of a fixation screen, a centrally presented face (“the student”), two peripheral card decks, and a lamp appeared simultaneously. With onset of the go-signal (lamp turning yellow), participants shifted their gaze to the left or right card deck (free-choice), thereby triggering a gaze shift of the central face 300 ms later either toward (in blocks with compatible action-effect mapping) or away from the targeted card deck (in blocks with incompatible action-effect mapping). In catch trials, the gaze response of the central face violated the current action-effect mapping, which participants should detect and signal with a manual keypress. In case of a no-go signal (lamp turning red, indicating enhanced attention on behalf of the casino to detect cheating), participants were instructed to withhold any (oculomotor and manual) response.

One female face photograph served as a stimulus and was available with eyes closed, direct gaze, and averted gaze (to the left/right). The size of the face ellipses amounted to 4.4° × 5.7° of visual angle (width × height), and faces were presented at the center of the screen. The picture of two card decks (5.3° × 3.5°) served as saccade targets. These saccade targets appeared at a distance of 8.8° of visual angle to the left and right of the central face. The picture of a lamp (3.5° × 3.1°) that was presented above the central face served as an imperative stimulus and was available in the following variants: light off, yellow light on, and red light on (see Fig. 1).

#### Procedure

The participants’ task was to saccade from the center of the screen to the card deck to the left or to the right. This saccade then triggered a gaze movement of the central on-screen face.

Specifically, each trial started with the presentation of a central white fixation cross (0.4° × 0.4°) on a black screen (see Fig. 1). After 700 ms, the central face (gaze directed at the participant), the two card decks, as well as the lamp (light off) appeared on the display. If a stable fixation on the face was detected, the lamp turned to one of two colors after a variable delay of 750 to 1,250 ms: Yellow indicated a go trial, whereas red indicated a no-go trial. In go trials, participants were instructed to shift their gaze as quickly as possible toward one of the two (left/right) card decks as soon as the yellow light appeared. They were told to choose the direction spontaneously in response to the go signal, but to look at each card deck about equally often and in no systematic order. In the instructions, it was emphasized that they should focus more on spontaneous decisions rather than on a perfectly even distribution of responses. However, if the distribution of choice frequencies of left- and rightward saccades deviated strongly from the instructed balanced distribution (more than 21 saccades into the same direction within a block of 33 trials), a reminder to choose each direction about equally often was presented after the block, and participants could proceed to the next block by pressing the space bar. In no-go trials, participants were instructed to withhold any oculomotor (and manual, see below) response.

Each saccade to the card decks triggered the central face to shift its gaze either to the left or to the right card deck, in accordance with the current action-effect mapping: In compatible blocks, the central face shifted its gaze to the looked-at card deck, whereas in incompatible blocks, the central face shifted its gaze to the opposite card deck. The delay between the landing of a target saccade within an interest area around one of the card decks and the onset of the gaze shift of the central face was 300 ms, a delay that yielded the most natural gaze following impression in pilot tests. The gaze shift of the central face and the card decks remained visible for 1,000 ms after the gaze shift. Then, the screen went blank for 1,000 ms before the next trial started (i.e., 2,000 ms after onset of the gaze shift). Participants were instructed to maintain their gaze on the chosen card deck until it disappeared. Crucially, if they shifted their gaze back to the central face while the action effect (gaze shift) was being displayed, the face would vanish, and a message would appear reminding them to keep their gaze on the selected card deck until the trial ended. If no saccade toward a card deck was detected within 1,500 ms after the go-signal, an omission feedback message was shown (1,000 ms). The next trial started immediately after the omission feedback had disappeared.

As already mentioned, we included catch trials in which the current action-effect mapping was violated, that is, in which the on-screen face looked into the opposite direction relative to the action-effect mapping of the current block. Participants were told to respond to these trials manually by pressing the space bar. If the space bar was pressed within 2,000 ms after the onset of the gaze shift, participants received an affirmative message; if no keypress occurred within 2,000 ms, an error message was displayed. In each case, the display went blank after 1,000 ms and a new trial started after another 1,000 ms. Additionally, an error message was displayed if the space bar was pressed in regular trials.

Participants encountered 264 trials of each action-effect mapping, resulting in a total number of 528 trials. Each mapping condition consisted of eight blocks with 33 trials each. Three catch trials and three no-go trials were presented within each block, with one catch trial and no-go trial randomly presented within a sequence of eleven trials. A calibration of the eye tracker was performed prior to each block using a five-point procedure. The experiment duration was around 60 min. The order of action-effect mappings was counterbalanced across participants: Half of the participants started with four blocks of the compatible action-effect mapping (compatible mapping = CM), followed by eight blocks of the incompatible action-effect mapping (incompatible mapping = IM), and finished with the remaining four blocks of the compatible action-effect mapping afterwards, resulting in the sequence CM – IM – IM – CM. For the other half of participants this sequence was reversed (IM – CM – CM – IM).

The experimental procedure was embedded in a casino-themed story. Participants were told that they would teach another person (visualized by an on-screen face) how to cheat in a card game. To do so, participants were told that they had to guide the attention of their student toward a specific card deck from which the next card should be drawn with the help of their eye movements. To indicate that the student had registered the teacher’s intention, the student would shift her gaze toward the card deck from which she would draw the next card. Participants were further told that the casino might become aware of such tactics, and in order to prevent that, they had agreed on two different strategies with their student: Looking at a card deck would mean to draw a card from the looked-at card deck for the first strategy (compatible action-effect mapping), whereas for the second strategy it would mean to draw a card from the opposite card deck (incompatible action-effect mapping). While the actual card drawing of the student was never displayed, the student’s gaze at the respective card deck was used as a proxy. Participants further received the information that the student (i.e., the on-screen face) would make mistakes from time to time (looking into the wrong direction, catch trials), and that their task was to indicate such erroneous behavior via a keypress (space bar).

Additionally, participants were instructed that the casino employees would look particularly closely at the gambling table via monitors from time to time. If this was the case, a warning will be displayed (lamp turning red) and neither an eye movement nor any other manual action must be executed by the participant (no-go trials). Participants could earn extra money for good performance if they detected the student’s mistakes and correctly withheld any gaze response in case of the red lamp.

### Results

#### Data treatment and analysis

Data and syntax for statistical analyses are publicly available on the Open Science Framework (https://osf.io/vw3na). Prior to analysis, all trials involving blinks (< 1%) or anticipatory saccades (latency < 60 ms) (< 1%) were removed, as well as all trials in which participants failed to initiate the requested saccade (6.6%) or in which the saccade did not land within the pre-defined areas around the card decks (a 200 × 200 pixel square – 7.0° x 7.0° of visual angle – around the center of the card decks; 4.2%). No-go trials were also excluded prior to analysis. This cleansing procedure resulted in the exclusion of 11.2% of all trials. Overall, the average proportion of leftward and rightward saccades was 49.1% and 50.9%, respectively, revealing a negligible (but significant) difference from an equal distribution in a two-sided *t*-test for dependent samples, *t*(103) = 2.05, *p* =.043, *d*_z_ = 0.20. In 3.1% of trials, saccades towards the center of the screen were detected after the onset of the action effect, causing the face to disappear and the corresponding warning message to be displayed.

#### Saccade latencies

##### Main analysis

**Fig. 2:**
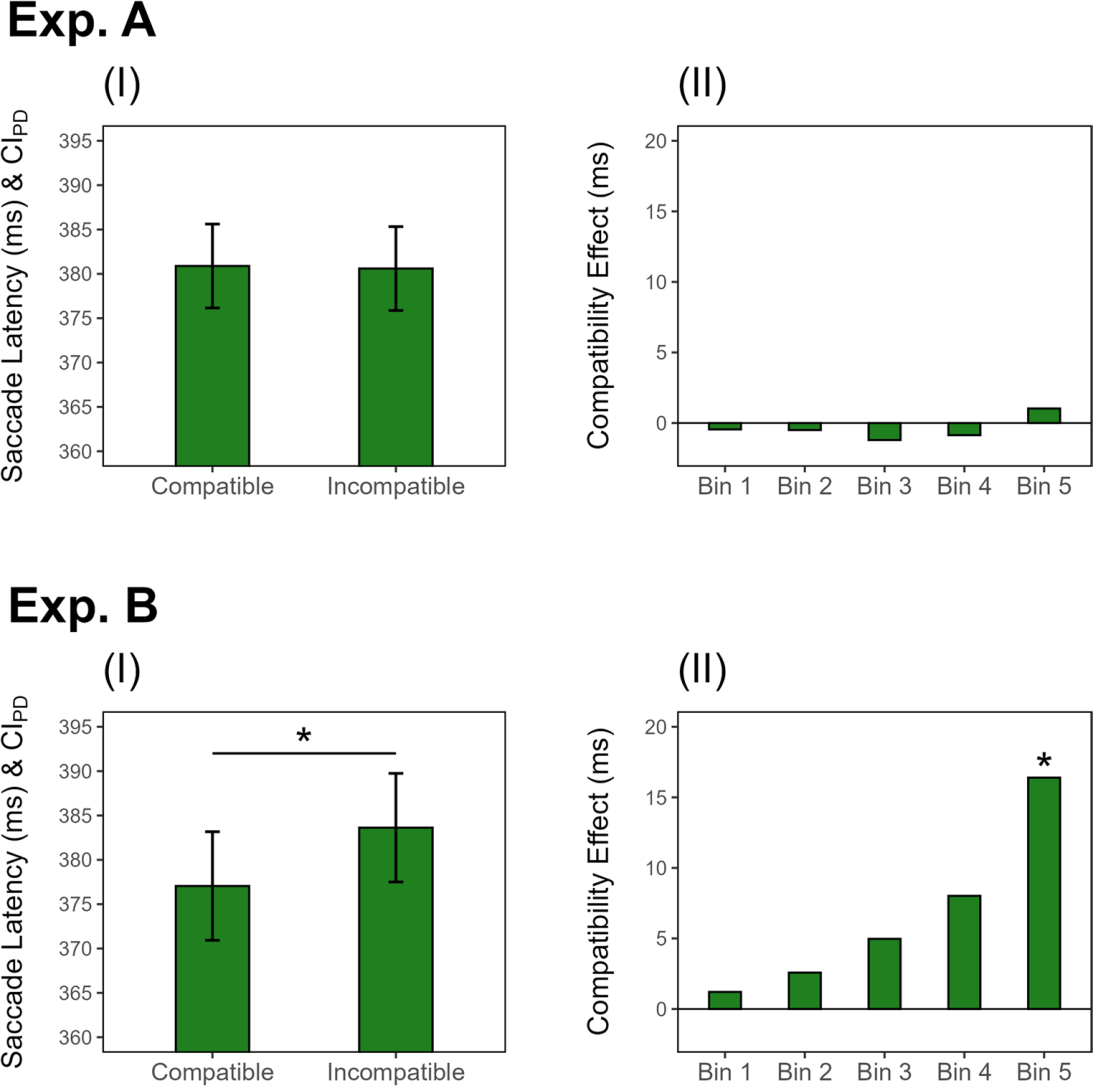
*Mean Saccade Latencies and Compatibility Effects in Experiments A and B. *(I) Mean saccade latencies (in ms) as a function of action-effect mapping (compatible vs. incompatible). Error bars depict the confidence intervals for the paired differences between compatible and incompatible condition (CI_PD_; Pfister & Janczyk, 2013). (II) Compatibility effects (in ms) calculated as mean saccade latency in the incompatible minus the compatible condition as a function of quintile bin, i.e., from fastest to slowest RTs. *: *p* < .05.

Saccade RT was defined as the interval between the onset of the yellow lamp and the initiation of a saccade with an amplitude of at least one third of the distance between the fixation cross and the center of the card deck in the present experiment. To test for the crucial difference between compatible and incompatible conditions, mean saccade RTs were analyzed with a two-tailed, paired sample *t*-test, revealing that saccades were initiated equally fast in the compatible condition (*M =* 381 ms, *SE* = 6.7) and the incompatible condition (*M =* 381 ms, *SE* = 7.1), *t*(103) = 0.12, *p =*.904, *d*_*z*_ = 0.01 (see Fig. 2).

##### Temporal characteristics

Although we did not find a compatibility effect, we followed our preregistered plan and analyzed temporal characteristics by performing a distribution analysis of the RTs. Specifically, we divided each participant’s RTs in the compatible and incompatible condition into five bins (from fastest to slowest RTs), ensuring that each bin contained approximately 20% of that participant’s trials per condition. The mean RTs within these bins were subjected to a repeated-measures analysis of variance (ANOVA) with bin (1 to 5) and compatibility (compatible/incompatible) as factors. To account for violations of the sphericity assumption, we report Greenhouse-Geisser corrected *p*-values and degrees of freedom. The analysis revealed no significant interaction between compatibility and bin, *F*(1.31, 134.64) = 0.11, *p* =.806, ƞ²_p_ <.01, as well as no significant main effect of compatibility, *p* =.869, in line with the main *t*-test analysis (see Fig. 2). As expected from the classification of the RTs according to duration, the main effect of bin was highly significant, *p <*.001.

Next, we also compared compatibility effects in the first and the second half of the experiment in a repeated-measures ANOVA with the factors half (first/second half) and compatibility (compatible/incompatible). This was possible because compatible (CM) and incompatible (IM) mapping blocks were presented in an ABBA-format, i.e., CM – IM – IM – CM, for one half of the participants, and IM – CM – CM – IM for the other half. The analysis revealed a significant main effect of experiment half with slower RTs in the first half of the experiment (*M =* 401 ms, *SE* = 7.2) compared to the second half (*M =* 361 ms, *SE* = 7.0), *F*(1, 103) = 118.46, *p* <.001, ƞ²_p_ =.54. The interaction of experiment half and compatibility was not significant, *F*(1, 103) = 1.08, *p* =.302, ƞ²_p_ =.01.

##### Mapping order

In line with our preregistration, we also analyzed the effect of mapping order, that is, whether the compatibility effect differed between those participants who encountered the compatible mapping first and those who encountered the incompatible mapping first using a mixed ANOVA with the between-subjects factor mapping order (compatible first/incompatible first) and the within-subjects factor compatibility (compatible/incompatible). The main effect of mapping order did not reach significance, *F*(1, 102) = 0.14, *p* =.706, ƞ²_p_ <.01. However, there was a significant interaction between compatibility and mapping order, *F*(1, 102) = 6.85, *p* =.010, ƞ²_p_ =.06. Two-tailed, paired sample *t*-tests showed that participants encountering the compatible mapping first had numerically faster reaction times in the incompatible (*M =* 380 ms, *SE* = 11.3) compared to the compatible condition (*M =* 387 ms, *SE* = 10.4), *t*(51) = 2.05, *p =*.045, *d*_*z*_ = 0.28. Conversely, participants who first encountered the incompatible mapping exhibited faster responses in the compatible (*M =* 375 ms, *SE* = 8.5) compared to the incompatible condition (*M =* 381 ms, *SE* = 8.8), although the difference did not reach significance, *t*(51) = 1.68, *p =*.100, *d*_*z*_ = 0.23. Taken together, apart from some negligible time-on-task effects, there was no indication of a REC effect whatsoever in the data.

#### Catch trial analysis

As an exploratory analysis, we also analyzed participants’ performance in the catch trials using two-tailed *t*-tests for dependent measures. Keypress latency in catch trials was not significantly different for the compatible (*M* = 619 ms, *SE* = 11.5) versus incompatible condition (*M* = 610 ms, *SE =* 11.7), *t*(103) = 1.47, *p* =.145, *d*_*z*_ = 0.14. Similarly, the error rate for catch trials did not significantly differ between the compatible (*M =* 7.2%, *SE* = 0.8) and incompatible condition (*M =* 8.5%, *SE =* 0.9), *t*(103) = 1.40, *p* =.164, *d*_*z*_ = 0.14.

### Discussion

In Experiment A, we compared RTs for producing (spatially) compatible versus incompatible action effects (i.e., gaze responses) in a peripherally visible target face. The results showed no significant response-effect compatibility effect. This may be due to the action effect being exclusively visible in the periphery, as we experimentally prevented participants from re-fixating the target face after their initial saccade in this experiment. Peripheral vision is inherently less sensitive than foveal vision for small changes (e.g., Rosenholtz, 2016; Strasburger et al., 2011), and while participants were clearly able to discriminate between gaze directions – as evidenced by the accurate detection of more than 90% of the catch trials – this additional difficulty may have interfered with regular action control and the formation of action-effect associations.

In addition, the restriction to revisit the face may have resulted in strong inhibitory demands associated with not looking at the crucial information (i.e., the target face). It appears reasonable to assume that participants would usually prefer to have the possibility to fixate the face. A saccade toward the face may even be made more likely since it is well known that saccades tend to be triggered by the sudden onset of movement (e.g., Abrams & Christ, 2003; Ludwig & Gilchrist, 2002) or due to an attempt to monitor the effects of their actions (e.g., Pfeuffer et al., 2016, 2023). To avoid setting off the warning message, participants may have been induced to primarily focus on suppressing gaze shifts back to the target face, thereby diverting attention away from the primary goal of leading the target face’s gaze. It is well known that the inhibition of prepotent saccades yield strong performance costs (Kürten et al., 2023a, b, 2024). Finally, not re-fixating the manipulated gaze of another person may prevent participants building up an accurate representation of the effects of their gaze behavior in the first place. Together, these factors may have prevented the occurrence of bindings (associations) between actions and effects here. As a point of comparison, we next present Experiment B, a version of the task in which we did not impose a physical constraint on revisiting the central face after effect onset. While we encouraged participants to maintain their gaze on the chosen deck of cards until the end of the trial (for the sake of comparison), we did not remove any visual information if participants were in violation of this rule.

## Experiment B

Experiment B (“unrestricted gaze”) followed a nearly identical procedure to Experiment A, with participants initiating spatially (in)compatible gaze shifts in a target face by directing their gaze to one of two objects. However, we did not impose any measures to prevent participants from re-fixating the target face after their initial saccade (which triggered the action effect). This setup created conditions more akin to a naturalistic, unrestricted gaze-leading scenario.

### Method

#### Stimuli and procedure

The experimental setup and procedure were almost identical to Experiment A: Participants were instructed to look at one of two card decks (positioned on the left or right), causing the gaze of a central face to either follow the participant’s gaze to the chosen card deck (compatible condition) or to shift to the opposite deck (incompatible condition), depending on the current action-effect mapping. As in Experiment A, participants were told to maintain their gaze on the selected card deck until the trial concluded. However, in the more “liberal” Experiment B, there were no technical restrictions regarding re-fixations: The face remained visible even if participants, contrary to instructions, shifted their gaze back to the central face displaying the action effect (i.e., gaze toward one card deck). In a post-survey at the end of the experiment, participants were asked to indicate the perceived difficulty of the catch trial task on a nine-point scale ranging from 1 (very easy) to 9 (very difficult).

#### Participants

A total of 62 participants were recruited (data collection in 2019). One participant was excluded from the analysis because of a technical failure during data collection, and another one due to unusually high RTs (> 3 *SD*s). Thus, data from 60 participants were analyzed (mean age = 24.5 years, *SD* = 4.1 years, age range: 18–36 years; 11 male). With this sample size, effect sizes of *d*_*z*_ ≥ 0.37 can be detected with a power of 1-β = 0.80 according to a post-hoc sensitivity analysis (G*Power, Faul et al., 2007). All participants provided informed consent prior to the start of the experiment and were compensated with either course credits or payment. They reported having normal or corrected-to-normal vision and were unaware of the study’s purpose. The study protocol and sample size were preregistered on the platform “aspredicted.org” before data collection began (see https://osf.io/4cebg).

### Results

#### Data treatment and analysis

 Data and syntax for statistical analyses are publicly available on the Open Science Framework (https://osf.io/4cebg). Before analysis, trials with blinks (< 1%), anticipatory saccades (< 1%), non-initiated saccades (7.0%), or saccades landing outside the predefined areas (3.5%) were excluded, along with no-go trials. This resulted in the removal of 10.8% of all trials. The average proportion of left- versus rightward saccades amounted to 49.1% versus 50.9% and did not differ significantly from an equal distribution, *t*(59) = 1.59, *p* =.118, *d*_z_ = 0.20, in a two-tailed, paired sample *t*-test.

#### Saccade latencies

##### **Main analysis**

In contrast to Experiment A, RTs significantly differed between trials with compatible and incompatible action effects in a two-tailed *t*-test for dependent measures, *t*(59) = 2.15, *p =*.036, *d*_*z*_ = 0.28: Saccades were initiated significantly faster in the compatible condition (*M =* 377 ms, *SE* = 8.8) compared to the incompatible condition (*M =* 384 ms, SE = 9.1) (see Fig. 2), indicating a response-effect compatibility benefit (see Janczyk et al., 2023; Kunde, 2001).

##### **Temporal characteristics**

To examine the characteristics of the compatibility effect, we conducted a repeated-measures ANOVA with the within-subjects factors bin (1–5, where each participant’s RTs were divided into five quantile-based bins per condition) and compatibility (compatible/incompatible). This analysis revealed a significant interaction between compatibility and bin, *F*(1.53, 90.28) = 4.07, *p* =.030, ƞ²_p_ =.06 (Greenhouse-Geisser corrected), indicating that the compatibility effect increased with longer RTs (see Fig. 2).

Pairwise *t*-tests (two-tailed, paired sample) revealed a significant effect of compatibility in the last bin (with slowest RTs), *t*(59) = 2.47, *p =*.016, *d*_*z*_ *=* 0.32, and a close to significant effect in the fourth bin, *t*(59) = 1.92, *p =*.060, *d*_*z*_ = 0.25, while no such effect was apparent in the remaining bins, all *p*s *>* .138. For the sake of completeness, the main effects from the ANOVA are reported: The main effect of bin was significant, *p <*.001, as expected from its computation, while the main effect of compatibility was also significant, *p* =.033, corroborating the *t*-test results from the main analysis.

Comparing compatibility effects in the first and the second half of the experiment, the repeated-measures ANOVA with the factors half (first/second half) and compatibility (compatible/incompatible) revealed a significant main effect of experiment half with slower RTs in the first half of the experiment (*M =* 393 ms, *SE* = 9.3) compared to the second half (*M =* 368 ms, *SE* = 9.0), *F*(1, 59) = 22.61, *p* <.001, ƞ²_p_ =.28, similar to Experiment A. The interaction of experiment half and compatibility was not significant, *F*(1, 59) = 2.18, *p* =.145, ƞ²_p_ =.04.

##### **Mapping order**

Neither the main effect of mapping order, *F*(1, 58) = 3.26, *p* =.076, ƞ²_p_ =.05, nor the interaction of compatibility and order of mapping, *F*(1, 58) = 1.45, *p* =.233, ƞ²_p_ =.02, reached significance in a mixed ANOVA with the between-subjects factor mapping order (compatible first/incompatible first) and the within-subjects factor compatibility (compatible/incompatible).

#### Catch-trial analysis

As in Experiment A, keypress latency in catch trials did not significantly differ between compatible (*M* = 611 ms, *SE* = 15.2) and incompatible action-effect mappings (*M* = 600 ms, *SE* = 16.3), *t*(59) = 1.26, *p* =.213, *d*_*z*_ = 0.16 (two-tailed *t*-test for dependent measures). The error rate also showed no significant difference between conditions with compatible (*M =* 5.2%, *SE* = 0.7) and incompatible action-effect mappings (*M =* 6.1%, *SE =* 0.8), *t*(59) = 0.92, *p* =.363, *d*_*z*_ = 0.12. In the post-experimental survey (9-point scale from 1 = very easy to 9 = very difficult), participants reported a mean perceived difficulty of the catch trial task of 3.34 (*SD* = 1.59; range: 1–8).

#### Exploratory analysis of refixations

For Experiment B, we conducted additional exploratory analyses to examine re-fixations on the central face after the action effect was initiated. Participants exhibited considerable variability in how often they looked back at the face during trials displaying the expected action effect (excluding no-go and catch trials), with refixation rates ranging from almost never (*Min* = 0.2% of trials) to nearly always (*Max* = 96.4%). The median proportion of refixations was 7.7%, indicating that most participants adhered well to the instruction to avoid returning their gaze to the center.^2^ To determine whether the compatibility effect observed in the main analysis was driven by participants who frequently perceived the action effect foveally, we reran the analysis excluding participants in the highest quartile of re-fixations (i.e., those with re-fixations in more than 22.2% of relevant trials). Even with these participants removed, the REC effect remained significant, *t*(44) = 2.43, *p* =.019, *d*_*z*_ = 0.36, and was numerically even slightly larger, with a 9 ms difference (compatible: *M* = 375 ms, *SE* = 10.1; incompatible: *M* = 384 ms, *SE* = 10.8) compared to the 7 ms difference in the main analysis.

### Discussion

Experiment B followed the same procedure as Experiment A, with one critical difference: We did not impose any control on whether participants re-fixated the target face after triggering the action effect with their initial saccade, merely instructing them to refrain from doing so. Overall, the results were comparable to Experiment A in terms of general RT level, while performance in catch trials was slightly better (5.6% vs. 7.8%). Crucially, we observed a small but significant REC effect in line with our expectations here: Participants were quicker to initiate saccades that triggered action effects spatially compatible with their saccade direction, with a (somewhat small but significant) 7 ms difference in RTs. This suggests that participants – in accordance with ideomotor theory – anticipated the effects their actions would produce, which in turn facilitated faster initiation of compatible saccades.

Notably, this pattern of results remained stable throughout the experiment, showing no significant change between the first and second half, and it was unaffected by the order in which participants encountered compatible or incompatible mappings. This consistency indicates that the effect was robust and not subject to learning or adaptation processes over time. Additionally, distribution analyses revealed that the expected trend (slower RT for incompatible vs. compatible trials) was numerically present in all RT bins. However, the difference was minimal in the lower bins and only reached statistical significance in the highest bin, corresponding to the slowest reaction times.

Regarding re-fixations of the central face (which we did not make impossible in this version of the experiment), participants showed considerable variability: While most participants only sporadically returned their gaze to the center after the action effect had occurred, some re-fixated the face in the majority of trials. Critically, the compatibility effect was still evident among those with relatively rare re-fixations, indicating that frequent foveal perception of the action effect was not required to exhibit the observed effect of response-effect compatibility.

## General Discussion

In the present study, we investigated the influence of anticipated gaze following behavior on saccade control. To this end, we asked participants to perform a directional saccade (action) which was consistently followed by another person’s eye movement (effect). The action-effect mapping differed between conditions, with gaze following behavior in the compatible condition and a gaze to the opposite side in the incompatible condition. While we observed no difference between compatibility conditions in Experiment A (“restricted gaze”), in Experiment B (“unrestricted gaze”) saccades were initiated faster in the compatible (vs. incompatible) action-effect mapping. Overall, this pattern of results aligns with the current state of research in multiple ways: first, by demonstrating the emergence of a response-effect compatibility (REC) effect in saccade control in a typical gaze leading/following situation, and second, by highlighting the context-sensitive nature of the effect. Disabling the possibility to fixate the action effect in Experiment A appeared sufficient to eliminate the effect (i.e., the build-up and/or utilization of associations between actions and effects), potentially reflecting increased cognitive demands or reduced processing of the action effects.

### Response-effect compatibility in a gaze leading scenario

The compatibility effect we observed in Experiment B is consistent with previous research by confirming that action-effect associations can be utilized for action control, not only in the manual domain (e.g., Janczyk et al., 2023; Kunde, 2001), but also in the oculomotor domain (Herwig & Horstmann, 2011; Huestegge & Kreutzfeldt, 2012; Riechelmann et al., 2017). However, in contrast to previous studies, we here established a situation in which participants were explicitly tasked with controlling another person’s gaze towards spatial locations using their own gaze. Crucially, gaze following influenced saccade latencies even though, in each trial, it occurred exclusively *after* the saccade had already been initiated. This is in line with the core ideomotor assumption of an endogenous activation of action effects prior to action generation. Specifically, anticipation of a leftward saccade of the on-screen face made it easier to initiate a leftward saccade, but harder to initiate a rightward saccade. Our findings are thus in agreement with the idea that anticipation of another person’s actions can shape saccade control. Moreover, this evidence bolsters the sociomotor framework, suggesting that basic ideomotor principles extend to social action effects, such as an interaction partner’s gaze direction (Kunde et al., 2018; Neszmélyi et al., 2022; Weller et al., 2020). Notably, the compatibility effect emerged even though the action effect was initially occurring in peripheral vision only, underscoring the flexibility of action-effect associations, which seem to incorporate not only foveal but also peripheral visual information to guide saccadic responses. In this context, it is not of primary importance that participants in Experiment B were, to some extent, able to glance back at the gaze follower’s face after its change in gaze direction: The actual *occurrence* of the effect (i.e., the onset of the gaze change) was always taking place extra-foveally, as is typical in a gaze leading/following situation.

Regarding temporal effect dynamics, distribution analyses usually demonstrate that REC effects are stronger with longer RTs (Keller & Koch, 2006; Kunde, 2001; Pfister et al., 2014). In Experiment B, we also observed stronger compatibility effects for slow responses. This is in line with the idea that in the case of longer RTs, there is more time for the time-consuming endogenous activation of the effect codes, eventually causing stronger interference with the codes of the actually required action (Kunde, 2001). A closer look at the nature of the corresponding interaction effect points toward some differences when comparing the results of the present oculomotor study to those of studies in the manual domain. In previous studies, compatibility effects were evident throughout all bins (or starting from the second bin) and increased with increasing RTs (Keller & Koch, 2006; Kunde, 2001; Pfister et al., 2013). In contrast, significant effects of compatibility in the present study were restricted to the last bin, even though a corresponding (non-significant) tendency was observed even in earlier bins. These differences with respect to previous experiments do not undermine the underlying rationale in general. However, they give rise to the presumption that compatibility effects might be less pronounced in the present setting, probably due to specific dynamics associated with saccade (vs. manual) control, such as the perhaps shorter planning process of saccades as compared to most manual actions.

### Small size and context-sensitivity of the spatial response-effect compatibility effect

While Experiment B demonstrated that compatibility between action and effect can significantly affect saccade latencies in a gaze-leading scenario, our overall data also raise several questions: Why was the compatibility effect in Experiment B rather small in size? And why did no compatibility effect emerge in Experiment A, despite its largely identical procedure when compared with Experiment B? A recent high-powered replication study of the REC effect in general with discrete manual responses and visual effects by Janczyk et al. (2023) revealed a reliable REC effect which, however, was smaller than in the original studies. As the authors discuss, REC is a fairly robust phenomenon when spatial effects are relevant for the movement task (Janczyk et al., 2015) or when responses and effects overlap regarding intensity (Kunde et al., 2004), or duration (Pfister et al., 2013), while they are sometimes smaller when action effects are nominally task-irrelevant and overlap with the action in terms of spatial features, as was the case in our study. Bearing this in mind, we will now explore several potential explanations for our observations.

First, a distinguishing feature of our experimental procedure – as we already discussed – is that the action effects did not initially occur at the participants’ fixation position (i.e., at the deck of cards), but in the periphery (i.e., within the central face), similar to a naturalistic gaze leading/following scenario. This could account for differences between compatibility effects in ideomotor saccade control compared to saccade control in situations where the effect onset is easier to perceive due to its foveal location (Herwig & Horstmann, 2011; Riechelmann et al., 2021). In general, the most important action effect of a saccade is the foveation of new information rather than the behavior that is triggered in other persons. Thus, an influence of anticipated behavior of the gaze follower (as visible in the periphery) on saccade control may be possible (as evidenced by our present results) but rather limited compared to the influence of the anticipated visual information at the fovea. The distinction between peripheral and foveal perception of the action effects is also highly relevant for the comparison between the two experiments. Given that the sole difference in procedure was that participants in Experiment A were experimentally restricted from re-fixating the target face once the action effect had been initiated, it would be plausible to speculate that perceiving the action effects exclusively in peripheral vision was not enough to form and utilize action-effect associations. Instead, (partial or potential) perception via the fovea might represent a necessary prerequisite for compatibility effects to emerge in a gaze-interaction scenario. However, explorative analyses of refixations in Experiment B indicated that the effect was still robust when excluding the quartile of participants with the highest proportion of refixations, suggesting that the mere possibility to look at the action effect might already suffice for the REC effect to emerge. As covert visual attention can be regarded as a precursor of overt eye movements (Deubel & Schneider, 1996), and given the highly interactive trans-saccadic integration processes at play (e.g., Stewart et al., 2020) participants may have attended and processed the faces more likely in Experiment B (in which refixations were allowed) than in Experiment A (in which refixation was discouraged). It is also possible that a few trials involving a refixation of the face promoted learning, as previous studies reported that trans-saccadic learning can be effective even after a few trials (Valsecchi et al., 2020).

Another potential reason for the differences in results between experiments as well as the overall weaker nature of the compatibility effect might stem from the dependence of the effect on specific task characteristics. Prior research has demonstrated that the emergence of ideomotor effects can be shaped by subtle differences in task setup, such as the action control mode adapted by participants (Pfister et al., 2010), attention toward the action effects (Janczyk et al., 2015), or the kind of stimuli used (Riechelmann et al., 2021). In the present study, the task setup and instructions limited (or in the case of Experiment A, eliminated) overt attentional monitoring through eye movements toward the effect (Pfeuffer et al., 2016), thereby possibly hampering the formation of strong action-effect associations. The experimental manipulation in Experiment A in particular may have been disruptive since it likely required participants to strongly inhibit any automatic saccades, which might in turn have altered their cognitive approach or primary focus when performing the task (especially considering that the eye region of faces is highly attention-grabbing, see Breil et al., 2022, and conveys a vast array of information, see Landmann et al., 2024). Specifically, it is well known that inhibiting saccades is a demanding cognitive task of its own (Kürten et al., 2023a, b).

In addition, studies suggest that on-screen faces can never fully establish a real social interaction (Rubo et al., 2020), likely reducing the saliency of action effects and thereby diminishing REC effects. Consistent with this, previous studies on sociomotor actions have reported effect sizes similar to the current (relatively small) effects when action effects were operationalized by videos or photos rather than by real-life interaction partners (Kunde et al., 2011; Pfister et al., 2017; see Weller et al., 2019 for a comparison of real-life and artificial interaction partners in an action-effect compatibility paradigm). Importantly, this explanation would suggest that the present study may underestimate the influence of gaze following behavior in real-life interactions.

Furthermore, specific features of our stimuli may have limited anticipated behavior effects compared with real-person interactions or typical inanimate action effects. Notably, the social effects in our study consisted solely of the movement of the student’s eyeball from straight gaze to averted gaze, producing less salient cues than the richer signals (eyelid movements, head or body turns) found in natural interactions. Anecdotal evidence from the post-experiment survey supports this interpretation: Although overall error rates were low (see Results), some participants reported difficulty detecting the gaze changes. It is therefore plausible that more salient social cues would modulate the extent to which anticipating social action effects shapes oculomotor behavior. To disentangle these factors, future studies should systematically (and ideally within-subject) manipulate the salience of the virtual partner’s response, for example, by varying the degree and presence of head turns, to identify which features of a gaze-following response are anticipated.

In contrast to most studies on action-effect compatibility (of both social and inanimate effects), we used a procedure in which participants could freely choose saccade direction (see Huestegge et al., 2019) rather than reacting to an imperative stimulus. Such free-choice procedures have been shown to slightly attenuate REC influence in the manual domain (Kunde, 2001, Experiment 3; but see Herwig & Horstmann, 2011). We chose the free-choice procedure because we assumed that participants would feel more involved in the interaction (as they actively had to choose where to guide their partner’s gaze). Additionally, it represents a method to rule out that observed REC effects are the result of an influence of acquired stimulus-effect-associations instead of action-effect associations (see Elsner & Hommel, 2001, and Kunde, 2001).

Research on effect monitoring has indicated that incompatible action effects are generally harder to monitor than compatible action effects, as reflected in higher dual-task costs (Wirth et al., 2018). This could also influence participants’ ability to detect catch trials, contributing to a more accurate detection while encountering compatible rather than incompatible action-effect mappings (see Pfister et al., 2017, for corresponding results). In the present study, however, we did not find any significant difference of catch trial detection between the compatible and the incompatible condition. This might signify that the task was not sensitive enough to detect any differences in effect monitoring (e.g., due to the low overall number of presented catch trials). In addition, visual action effects are usually monitored by eye movements toward the effect location (Pfeuffer et al., 2016). As the present setting and instructions prevented such overt attentional monitoring processes to some degree, this may have further attenuated corresponding effects.

### Constraints on generality

Regarding the generalizability of our findings, it is important to note that while the two experiments had comparable sampling demographics, the majority of participants in both cases were young adult females, many of whom were university students. Therefore, the present findings may not be fully generalizable to other populations, for example adolescents or older adults who might exhibit age-related differences in action control processes. Additionally, our study used only a single face stimulus – a frontal view of a woman with a clearly visible eye area. As a result, we cannot be certain whether our findings would generalize to other faces where gaze shifts might be less or more salient, for example, due to differences in facial features (e.g., eye size, wrinkles) or variations in angle or lighting. Finally, the artificial context of the interaction established in our design does not fully capture the dynamics and complexity of realistic face-to-face interactions. While this controlled approach was necessary to isolate and examine the specific processes of interest, it limits the generalizability to real-life social settings.

However, our central conclusion here is that basic ideomotor processes are also at play in a context involving social stimuli. As such, it does not require the assumption that our experimental setup simulated a fully realistic social interaction. In fact, it appears very likely that the same ideomotor processes that govern action control in inanimate environments are also relevant in the social domain. Specifically, similar ideomotor effects have been reported in inanimate (Huestegge & Kreutzfeldt, 2012) and in social (Herwig & Horstmann, 2011) situations. However, our present results demonstrate for the first time that ideomotor mechanisms are also at play in gaze leading/following situations, in which the effect onset occurs well outside of the central foveal region.

## Conclusion

In summary, the present results suggest that in a social (gaze-based) interaction scenario, people can control the gaze of others by anticipating the other’s gaze responses to their own gaze, in line with the ideomotor framework, even if the effect is initially perceived in the visual periphery as is typical in a gaze leading/following situation. However, the corresponding effect appears to be small and context-sensitive. Nevertheless, the results are theoretically important as they further emphasize the potential relevance of another person’s behavioral responses for action control of even basic (oculomotor) actions.

## Data Availability

The preregistrations, along with all data and analysis scripts are openly available on the Open Science Framework (Experiment A: https://osf.io/vw3na; Experiment B: https://osf.io/4cebg).

## Footnotes

1 For a better understanding, we decided to present the stricter, more controlled version of the experiment (no foveation of the partner’s gaze response possible) first, even though the respective data were collected later.

2 Ideally, analyses would have focused on trials preceding the first refixation; however, because these refixations largely occurred during the task familiarization phase, such a subset could not be meaningfully extracted.
